# External Validation of the Early Prediction of Functional Outcome After Stroke Prediction Model for Independent Gait at 3 Months After Stroke

**DOI:** 10.3389/fneur.2022.797791

**Published:** 2022-05-02

**Authors:** Janne M. Veerbeek, Johannes Pohl, Jeremia P. O. Held, Andreas R. Luft

**Affiliations:** ^1^Department of Neurology, University of Zurich and University Hospital Zurich, Zurich, Switzerland; ^2^Neurocenter, Luzerner Kantonsspital, Lucerne, Switzerland; ^3^Department of Rehabilitation Sciences, KU Leuven – University of Leuven, Leuven, Belgium; ^4^Rehabilitation Center Triemli Zurich, Valens Clinics, Zurich, Switzerland; ^5^Cereneo, Center for Neurology and Rehabilitation, Vitznau, Switzerland

**Keywords:** stroke, prognosis, external validation, gait, lower extremity, logistic model, outcome, rehabilitation

## Abstract

**Introduction:**

The Early Prediction of Functional Outcome after Stroke (EPOS) model for independent gait is a tool to predict between days 2 and 9 poststroke whether patients will regain independent gait 6 months after stroke. External validation of the model is important to determine its clinical applicability and generalizability by testing its performance in an independent cohort. Therefore, this study aimed to perform a temporal and geographical external validation of the EPOS prediction model for independent gait after stroke but with the endpoint being 3 months instead of the original 6 months poststroke.

**Methods:**

Two prospective longitudinal cohort studies consisting of patients with first-ever stroke admitted to a Swiss hospital stroke unit. Sitting balance and strength of the paretic leg were tested at days 1 and 8 post-stroke in Cohort I and at days 3 and 9 in Cohort II. Independent gait was assessed 3 months after symptom onset. The performance of the model in terms of discrimination (area under the receiver operator characteristic (ROC) curve; AUC), classification, and calibration was assessed.

**Results:**

In Cohort I [*N* = 39, median age: 74 years, 33% women, median National Institutes of Health Stroke Scale (NIHSS) 9], the AUC (95% confidence interval (CI)] was 0.675 (0.510, 0.841) on day 1 and 0.921 (0.811, 1.000) on day 8. For Cohort II (*N* = 78, median age: 69 years, 37% women, median NIHSS 8), this was 0.801 (0.684, 0.918) on day 3 and 0.846 (0.741, 0.951) on day 9.

**Discussion and Conclusion:**

External validation of the EPOS prediction model for independent gait 3 months after stroke resulted in an acceptable performance from day 3 onward in mild-to-moderately affected patients with first-ever stroke without severe prestroke disability. The impact of applying this model in clinical practice should be investigated within this subgroup of patients with stroke. To improve the generalizability of patients with recurrent stroke and those with more severe, neurological comorbidities, the performance of the EPOS model within these patients should be determined across different geographical areas.

## Introduction

Recovery of gait is one of the main priorities in motor stroke rehabilitation (1). Knowledge regarding the prognosis of gait outcome is an integral part of clinical decision-making, allows stratifying patients for (research) interventions, can improve rehabilitation efficacy, and aids in properly informing patients and their relatives (2). Various prediction models for independent gait after stroke have been developed, and the Early Prediction of Functional Outcome after Stroke (EPOS) prediction model (3) has been indicated to be one of the most promising models (4, 5).

In 2011, the EPOS prediction model for regaining independent gait 6 months after stroke was developed in patients with first-ever ischemic stroke who were not able to walk independently within the first 72 h after symptom onset (3). The Functional Ambulation Categories (FAC) (6) was the selected measurement instrument to capture the gait abilities of a patient and based on which ‘independent gait' was defined as a score of ≥4/5 and ‘dependent gait' as a score of <4/5 (3). The multivariable logistic model contained two predictors: sitting balance as assessed by the Trunk Control Test (TCT-s) (7) and strength of the paretic lower limb, measured by the Lower Extremity subscale of the Motricity Index (MI-LE) (7). Patients who were able to sit unsupported for 30 s (TCT-s score of 25/25) and had some strength in the paretic leg (MI-LE score of ≥25/100) within 72 h post-stroke had a probability of 97% to walk independently 6 months later (3). The probability of patients not fulfilling these criteria was 27% at baseline (<72 h post-stroke) and decreased to 10% when the predictors were retested on day 9.

Although the EPOS model seems to be feasible to apply in clinical practice and its performance is encouraging, the model has not been externally validated until now. However, external validation is an important step toward the clinical application of prediction models. With external validation, the reproducibility and generalizability of the model, in an independent patient sample with a different case-mix, are tested by determining the model's level of overfitting and performance within this new sample (2, 8, 9). External validation is needed, as it has been shown that the performance of prediction models in an independent sample is often lower than that in the sample in which the model was developed (10).

The EPOS prediction model as developed in 2011 did use a 6-month endpoint for independent gait. However, 6-months might be too conservative, as most behavioral change takes place within the first week to the first few months (11). When looking at gait specifically, Kennedy and colleagues recently showed that the median time needed to walk 50 m without assistance was 6 (Q1 = 2, Q3 = 63) days and that by 3 months, 75% of the patients were able to walk 50 m unassisted (12). The percentage of 75% did not differ much from the 79% of patients who walked independently at 6 months in the EPOS study in 2011, suggesting that not many patients regained independent gait between 3 and 6 months after stroke. This emerged knowledge supports investigating the applicability of the EPOS model for the outcome of gait 3 months after stroke onset instead of the original 6 months.

Therefore, we aimed to perform a temporal and geographical external validation of the EPOS prediction model for independent gait after stroke (3) in two cohorts in Switzerland. However, contrary to the endpoint of 6 months post-stroke in the original study, the EPOS prediction model was externally validated for a 3-month endpoint. We hypothesized that the performance of the model in the validation cohorts would be acceptable but lower than that in the development cohort (10).

## Materials and Methods

### Design

We carried out a secondary data analysis of two prospective longitudinal observational studies. Recruitment for study 1 (Cohort I) took place from 09/2017 until 11/2019 and the study included three visits. Visit 1 took place within 48 h, visit 2 on day 7 ± 2, and visit 3 on day 90 ± 7 after symptom onset. Study 2 (Cohort II) started on 09/2018 and ended on 03/2021, for this work relevant study visits included days 3 ± 2 (visit 1), 10 ± 2 (visit 2), and 90 ± 7 (visit 3) post-stroke. For both studies, patients consecutively admitted to the stroke unit of the Department of Neurology of the University Hospital Zurich (Switzerland) and diagnosed with a stroke were screened. Ethical approval from the cantonal ethics committee Zurich was obtained before the start of the studies (BASEC identifiers 2017-00889 and 2017-01070), and both studies were prospectively registered at ClinicalTrials.gov (Identifiers NCT03287739 and NCT03522519). The same ethical committee approved secondary data analysis (BASEC identifier 2020-00218). Reporting was done according to the Strengthening the Reporting of Observational Studies in Epidemiology (STROBE) (13) and Transparent Reporting of a multivariable prediction model for Individual Prognosis or Diagnosis (TRIPOD) statements (14).

### Patients

Study characteristics for Cohorts I and II are described in Table 1. At the University Hospital Zurich, the median length of hospital stay for stroke patients was 6 (Q1 = 2, Q3 = 11) days, and patients typically received 45–75 min of individualized physical and occupation therapy per day during their stay at the acute stroke unit 5 days a week. All patients of Cohort I were discharged to an inpatient rehabilitation center. In Cohort II, 77 out of 78 patients (99%) received inpatient stroke rehabilitation, and one patient did not receive any rehabilitation after hospital discharge. In both cohorts, patients were treated according to the current national guidelines (15) and local protocols. Motor rehabilitation was patient-centered and was applied with a task-oriented approach with repetitive nature.

**Table 1:** Key characteristics of the development and validation studies.

**Characteristic**	**Development cohort (3) (*N* = 154)**	**Validation cohort I (*N* = 39)**	**Validation cohort II (*N* = 78)**
Recruitment period	02/2007–11/2009	10/2017–11/2019	09/2018–12/2020
Setting	9 acute hospital stroke units in the Netherlands	1 acute hospital stroke center in Switzerland	1 acute hospital stroke center in Switzerland
Inclusion criteria	(1) First-ever ischemic anterior circulation stroke (2) ≥18 years (3) Mono- or hemiparesis <72 h (4) Premorbid Barthel Index ≥19 (5) No severe deficits in communication, memory, or understanding (6) Written informed consent (7) FAC <4 within 72 h	(1) First-ever unilateral ischemic stroke <48 h, confirmed by MRI-DWI and/or CT (2) ≥18 years (3) NIHSS arm ≥1 (4) Prestroke modified Rankin Scale ≤2 (5) Able to follow one-staged commands (6) Informed consent after participants' information	(1) First-ever ischemic or hemorrhagic stroke, confirmed by MRI-DWI and/or CT (recurrent strokes are allowed when already included in this study after a first-ever stroke) (2) ≥18 years (3) Motricity Index (sum of the upper and lower extremity subscales) <200 (4) Prestroke modified Rankin Scale ≤2 (5) Written informed consent of the patient or its legal representative after participants' information
Exclusion criteria	Not formulated	(1) Neurological or other diseases affecting the upper limb(s) before stroke (2) Intravenous line in the upper limb(s) that limited assessment (3) Contra-indications on ethical grounds (4) Expected or known non-compliance, severe drug and/or alcohol abuse (5) For the current work: FAC ≥4 within 48 h	(1) Neurological or other diseases affecting upper limb use and/or physical activity before stroke (2)Contra-indications on ethical grounds (vulnerable persons) (3) Known or suspected non-compliance, drug and/or alcohol abuse (4) For the current work: FAC ≥4 at day 3 ± 2
Outcome	FAC: <4 vs. ≥4; 6 months post-stroke	FAC: <4 vs. ≥4; 3 months post-stroke	FAC: <4 vs. ≥4; 3 months post-stroke
Predictors^*^	TCT-s: <25 vs. 25; days 2, 5, and 9 post-stroke MI-LE: 25 vs. ≥25; days 2, 5, and 9 post-stroke	TCT-s: <25 vs. 25; days 1 and 8 post-stroke MI-LE: 25 vs. ≥25; days 1 and 8 post-stroke	TCT-s: <25 vs. 25; days 3 and 9 post-stroke MI-LE: 25 vs. ≥25; days 3 and 9 post-stroke

* *, dichotomized predictors are coded 0 and 1; CT, Computed Tomography; FAC, Functional Ambulation Categories; MI-LE, Motricity Index – Lower Extremity subscale; MRI-DWI, Magnetic Resonance Imaging – Diffusion Weighted Imaging; NIHSS, National Institutes of Health Stroke Scale; TCT-s, Trunk Control Test – Sitting Balance item*.

### Data Collection

Trained, unblinded physical therapy researchers performed assessments. The predictors were collected during the hospital stay. The outcome of 3 months post-stroke was assessed during an outpatient visit at the hospital or at the patient's place of residence.

### Outcome

The dependent variable was independency in gait 3 months after stroke as measured by the Functional Ambulation Categories (FAC) (6, 16, 17). The FAC is an observational scale that classifies the walking ability of a patient, regardless of whether walking aids or orthoses are used. The FAC-score ranges from 0 to 5, with higher scores indicating a better walking ability. FAC was dichotomized into ≥4/5 points (independent gait; favorable outcome) and <4/5 points (dependent gait; unfavorable outcome) (3). A score of 4 means that the patient is able to walk on level surfaces, while with a score of 5, the patient can walk independently anywhere.

### Predictors

The independent variables included the sitting balance item of the Trunk Control Test (TCT-s, score range 0–25) (7) and the lower extremity subscale of the Motricity Index (MI-LE, score range 0–100) (7) as measured during study visits 1 and 2. According to the EPOS prediction model, the TCT-s was dichotomized into 25/25 (1, sitting balance present) vs. <25/25 points (0, sitting balance absent). The MI-LE was dichotomized into ≥25/100 (1, strength present) vs. <25/100 (0, strength absent).

Data additionally collected to characterize the patients of the validation cohorts to allow comparison with the development cohort and judge whether the results are applicable to patients seen in clinical practice included patient demographics, stroke characteristics, the National Institutes of Health Stroke Scale (NIHSS) (18, 19), the Fugl-Meyer Motor Assessment (20), and the modified Rankin Scale (mRS) (21).

### Differences Between Development and Validation Studies

Key characteristics of the development study (3) and the two validation studies are presented in Table 1.

### Sample Size

This is a secondary data analysis and, therefore, no sample size calculation was performed.

### Statistical Analysis

Patients who died before the day-90 visit were excluded from the analysis. Baseline characteristics of the included patients were analyzed by nonparametric descriptive statistics. The EPOS model for independent gait was developed for making a prediction at days 2, 5, and 9 post-stroke (3). The model for day 2 was validated using the data from visit 1 and the model at day 9 was validated with the data from visit 2. As none of the studies had a measurement time point nearing day 5 post-stroke, the model at day 5 was not externally validated.

Differences between patients with and without missing data on predictors and/or outcome were formally tested with the Mann–Whitney *U*-test or chi-squared test. Missing data on predictors and outcomes were imputed by the R package ‘Multivariate Imputation by Chained Equations' (mice) with 100 imputations and 5 iterations (22). Data used for imputation included the raw values of the TCT-s at visits 1 and 2, MI-LE at visits 1 and 2, FAC at visit 3, the NIHSS total score at visit 1, age, gender (women/men), affected side (left/right), and the NIHSS items consciousness and hemianopia at visit 1 (23). After imputation, patients without an outcome were excluded (24, 25). The imputed data were used for the primary analysis. In addition, the analysis with raw data was presented.

The following equations were used to describe the probabilities of achieving independent gait 3 months post-stroke:


$$\begin{array}{l} \text{Day }2:P=1/1+(exp^{\left[-(-0.982\;+\;2.691{\;}^{*}\text{TCT}-\text{s }+\;2.083{\;}^{*}\text{MI}-\text{LE})\right]})\\ \;(\text{presence}=1,\text{absence}=\;0) \end{array}$$

$$\begin{array}{l} \text{Day }9:P=1/1+(exp^{\left[-(-2.226\;+\;3.629{\;}^{*}\text{TCT}-\text{s }+\;1.854{\;}^{*}\text{MI}-\text{LE})\right]})\\ \;(\text{presence}=1,\text{ absence}=\;0) \end{array}$$

Discrimination of the EPOS model was determined based on the area under the receiver operating characteristic (ROC) curve (AUC) with its 95% confidence interval (CI), with an AUC of >0.75 reflecting a clinically useful model (26). The classification was assessed by the sensitivity, specificity, and positive and negative predictive values. Calibration plots visualized the agreement between predicted and observed probabilities. RStudio version 3.6.3 was used for all analyses (27) and a value of *P* < 0.05 was considered to be statistically significant.

## Results

**Figure 1:**
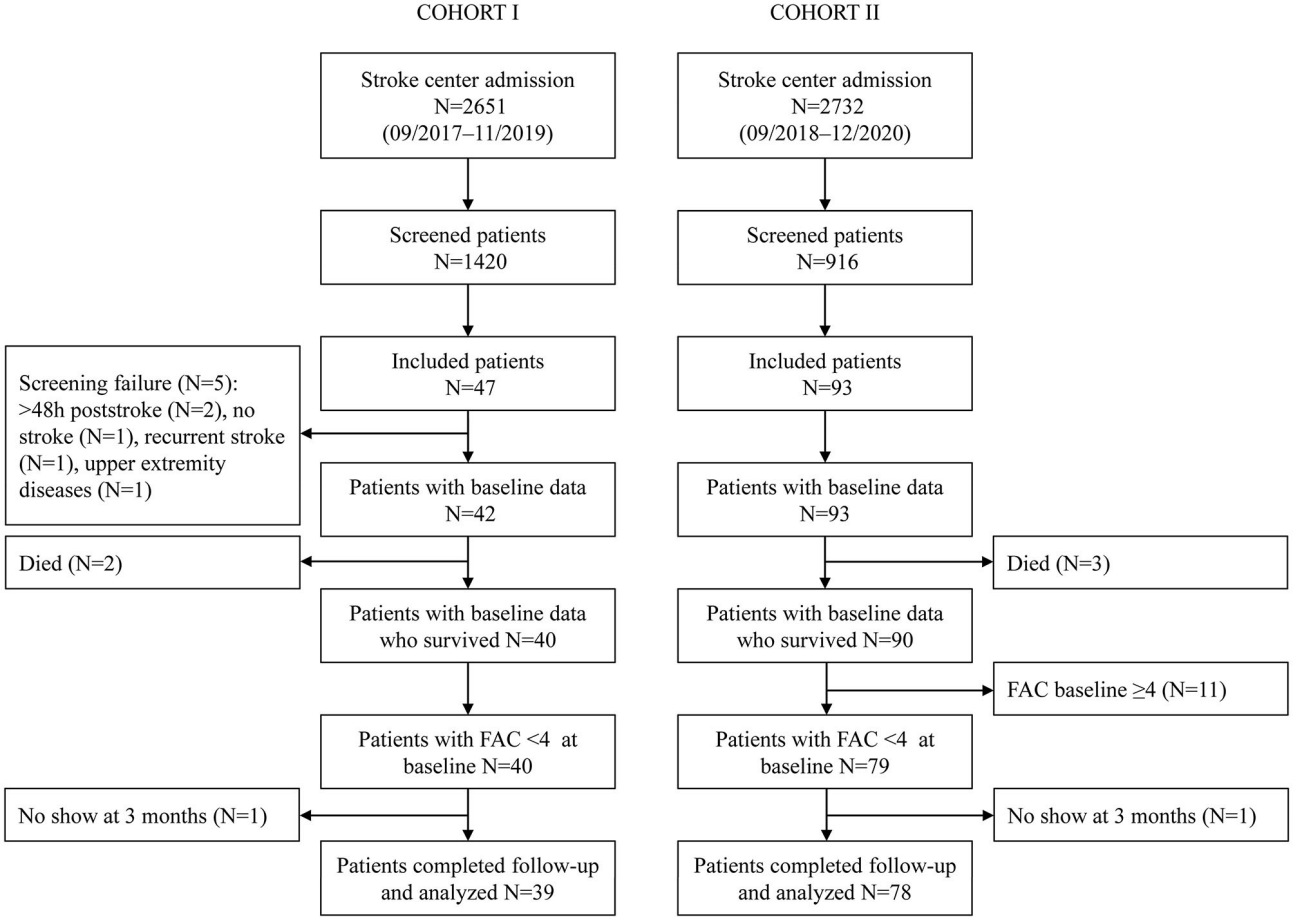
Patient flowchart.

In total, 39 non-ambulatory patients were analyzed in Cohort I and 78 in Cohort II (Figure 1). Patients in Cohort I had a median (Q1–Q3) age of 74 (69–77) years, 13 (33.3%) were women, 13 (33.3%) had suffered a left hemispheric stroke, and the baseline NIHSS amounted to 9 (5.5–13.5). Patients included in Cohort II had a median (Q1–Q3) age of 69 (60–77) years, 29 (37.2%) were women, 60 (76.9%) had an ischemic stroke, 37 (47.4%) had a lesion in the left hemisphere, and the median baseline NIHSS was 8 (5–12). Visit 1 took place on day 1 post-stroke in Cohort I and on day 3 in Cohort II. The second visit in Cohorts I and II was performed on days 8 and 9, respectively. In Cohort I, 17 (43.6%) patients had a TCT-s of 25/25, and 28 (71.8%) patients had an MI-LE score of ≥25/100 at visit 1. In Cohort II, 47 (60.3%) had a TCT-s of 25/25, and 60 (76.9%) an MI-LE score of ≥25/100 at visit 1. In validation of Cohorts I and II, 71.8 and 74.4% of the patients were able to walk independently at 3 months post-stroke, respectively. The patient characteristics of the validation cohorts are described in more detail in Supplementary Table 1 and can be compared with the development cohort that is described in the same table. Early changes in the walking ability are described in Supplementary Tables 2, 3 for Cohorts I and II, respectively. At the second study visit, 10 patients in Cohort I and 22 patients in Cohort II walked independently.

In both validation cohorts, full data on FAC outcomes were available in the analyzed samples. In Cohort I, the following data on predictors were missing: MI-LE visit 1 (*N* = 1), TCT-s visit 1 (*N* = 2), and MI-LE visit 2 (*N* = 3). For Cohort II, there were missing data for visit 2 for both the TCT-s and MI-LE (*N* = 4 each). Patients with missing data did not significantly differ from those without missing data (Supplementary Table 4).

**Figure 2:**
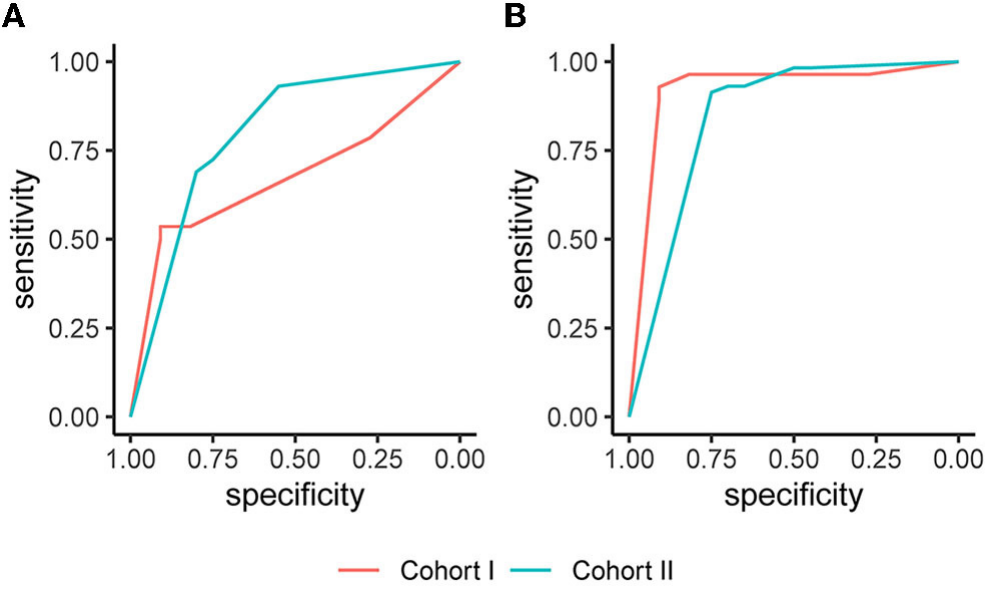
Receiver operating characteristic (ROC) curves based on the imputed datasets. **(A)** The Early Prediction of Functional Outcome after Stroke (EPOS) model for day 2, measured on day 1 in Cohort I and on day 3 in Cohort II; **(B)** The EPOS model for day 9, measured on day 8 in Cohort I and day 9 in Cohort II.

In Cohort I, analysis of the imputed data resulted in an AUC (95% CI) of 0.675 (0.510, 0.841) on day 1 and 0.921 (0.811, 1.000) on day 8. In Cohort II, the AUC (95% CI) amounted to 0.801 (0.684, 0.918) on day 3 and 0.846 (0.741, 0.951) on day 9. The corresponding ROC curves are displayed in Figure 2. The sensitivity in Cohort I increased from 0.786 on day 1 to 0.964 on day 8, and the specificity improved from 0.273 on day 1 to 0.727 on day 8. In Cohort II, the sensitivity was 0.931 on days 3 and 9, and the specificity values were 0.550 on day 3 and 0.650 at day 9. Full information on the discrimination and classification measures of the EPOS model in the two independent cohorts are reported in Table 2.

**Table 2:** Discrimination of the Early Prediction of Functional Outcome after Stroke (EPOS) model for independent gait in the development and validation cohorts based on imputed datasets.

	**Development**	**Validation**	**Validation**
	**cohort (3)**	**cohort I**	**cohort II**
**Model day 2**	*N* = 154	*N* = 39	*N* = 78
Accuracy (95% CI)	0.889	0.641	0.833
Sensitivity	0.926	0.786	0.931
Specificity	0.750	0.273	0.550
Positive predictive value	0.933	0.733	0.857
Negative predictive value	0.727	0.333	0.733
No information rate	N/R	0.718	0.744
*P*-Value (Acc > NIR)	N/R	0.892	0.041
AUC (95% CI)	N/R	0.675	0.801
		(0.510, 0.841)	(0.684, 0.918)
**Model day 9**	*N* = 154	*N* = 39	*N* = 78
Accuracy (95% CI)	0.916	0.897	0.859
Sensitivity	0.959	0.964	0.931
Specificity	0.750	0.727	0.650
Positive predictive value	0.936	0.900	0.885
Negative predictive value	0.828	0.889	0.765
No information rate	N/R	0.718	0.744
*P*-Value (Acc > NIR)	N/R	0.007	0.010
AUC (95% CI)	N/R	0.921	0.846
		(0.811, 1.000)	(0.741, 0.951)

*Acc, accuracy; AUC, area under the curve; CI, confidence interval; N/R, not reported; NIR, no information rate*.

**Figure 3:**
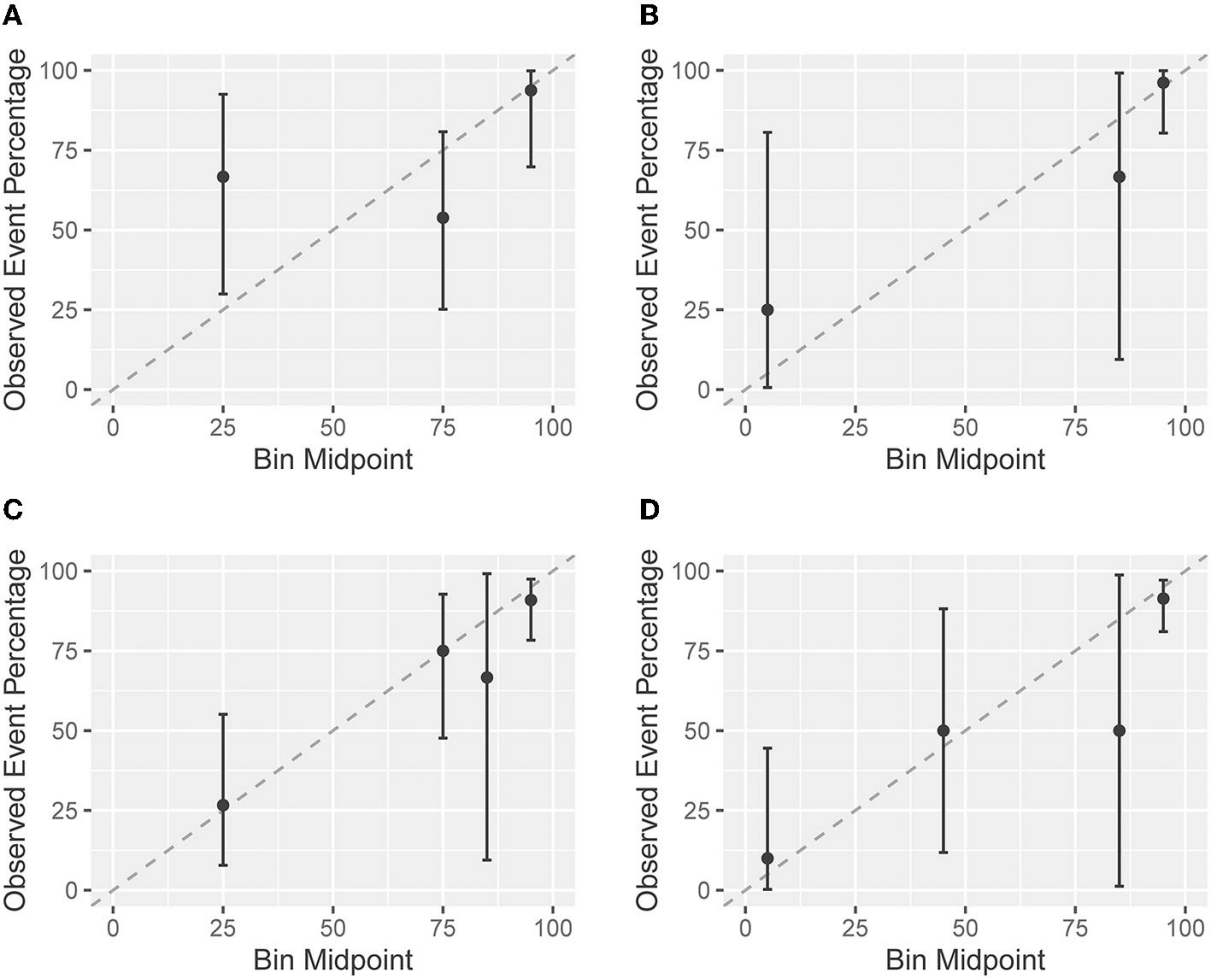
Calibration plots based on the imputed datasets. A calibration plot shows the agreement between the predicted probabilities based on the Early Prediction of Functional Outcome after Stroke (EPOS) model for independent gait on the *x*-axis and the in the observed probabilities of validation cohorts on the *y*-axis. The closer the points are to the plotted diagonal line, the better the calibration. Points above the diagonal line indicate that the model is rather pessimistic, points below the line indicate that the model is rather optimistic. Confidence intervals (CIs) overlapping with the diagonal 45 degree line indicate no significant difference between predicted and observed probabilities. Cohort I: **(A)** the EPOS model for day 2, measured on day 1 post-stroke; and **(B)** the EPOS model for day 9, measured on day 8 post-stroke. Cohort II: **(C)** the EPOS model for day 2, measured on day 3 post-stroke; and **(D)** the EPOS model for day 9, measured on day 9 post-stroke.

The agreement between predicted and observed probabilities is presented in the calibration plots of Figure 3. For patients in Cohort I who had a low probability of regaining independent gait as predicted by the EPOS model on day 1, the predicted probability was lower than the observed probability (Figure 3A). On day 8, the difference between predicted and observed probabilities for patients with a low predicted probability was no longer significant (Figure 3B). For patients with a higher predicted probability of regaining independent gait, the observed probabilities matched the predicted probabilities on both days (Figures 3A,B). In Cohort II, the predicted and observed probabilities did not significantly differ for both days 3 and 9 (Figures 3C,D).

The results of the analysis based on the raw data are reported in Supplementary Table 3 and Supplementary Figure 1 and did not show differences.

## Discussion

This is the first external validation of the EPOS model for predicting independent gait after stroke, marking an essential step toward clinical implementation. An important difference with the original study is that the endpoint was assessed 3 months after symptom onset instead of 6 months. Even though the timing of the endpoint was earlier, the temporal and geographical external validation showed an adequate performance on days 3, 8, and 9, but not on day 1 post-stroke. The discriminative ability of the EPOS model on day 3 was good and further increased when predictors were retested on day 8 or 9. In analogy to this, the positive predictive and negative predictive values were high on days 3, 8, and 9, with the positive predictive value slightly outperforming the negative predictive value at both time points. The agreement between predicted and observed probabilities was good, although CIs were relatively wide, except for patients with a low predicted probability on day 1. For these patients, the observed probability was significantly higher than the predicted probability.

The performance of the EPOS model for independent gait from day 3 onward matches the performance that was found in the development study, even though the endpoint in the validation cohorts was 3 months instead of the 6-month endpoint that was applied in the development study. Furthermore, the validation cohorts had a different case-mix and were recruited in another country with a different healthcare system (Switzerland vs. the Netherlands). With that, this external validation study shows that the EPOS model can be applied to predict outcomes at 3 months after stroke. This finding supports the assumption described in the introduction that, when patients regain independent gait after stroke, they do so within the first 3 months after stroke, and there are hardly any patients who shift from ‘dependent gait' to ‘independent gait' between 3 and 6 months. This matches the earlier finding of Baer and Smith, that patients with stroke achieve independent gait at a mean [standard deviation (SD)] of 16.0 (29.7) days (28). Our data furthermore showed that a proportion of patients shift from ‘dependent' to ‘independent' within a week after the first assessment of predictors: 27% of the patients in Cohort I and 30% in Cohort II achieved a FAC of ≥4/5 at the second study visit. Although in all cohorts patients with first-ever stroke who were independent before their stroke were included, there are differences between the development and validation cohorts, such as the lower proportion of women included in the validation cohorts, a higher median age in Cohort I, the inclusion of patients with hemorrhagic stroke in Cohort II, and the prevalence of thrombectomy as an acute medical treatment in both validation cohorts. This supports the generalizability of the EPOS model to a wider stroke population than that included in the development study. Additionally, this study highlights that an application of the EPOS model on day 1 post-stroke seems to be invalid as the low specificity and low negative predictive value indicate that the model is too pessimistic at this early time point. This could be explained by the dynamics of neurological functions in the (hyper)acute phase in which a portion of the patients show a quick neurological recovery (29), or masking of the patient's ability by, e.g., vertigo, hypotension, or fatigue. It could also be attributable to the fact that patients were not or not often mobilized before the TCT-s was performed, and thus, the test score represents one of their first attempts to sit at the edge of the bed.

This first external validation of the EPOS model for independent gait is promising, and although the model can be applied to a defined subgroup of patients with stroke, there are steps that need to be taken before the model is ready for a large-scale implementation. Validation studies are needed that preferably recruit a large and heterogeneous group of patients at different sites, such as those with recurrent strokes, other neurological diseases, and more comorbidities. These studies should not only be performed in western European countries but also in geographical regions with different healthcare systems. On a smaller scale, both the impact and the implementation of applying the EPOS model in clinical practice should be investigated. Although it is assumed that the application of prediction models has several benefits for the healthcare system, including increased efficiency through stratified medicine, it remains unknown how the application of the EPOS model affects these aspects. No impact studies are available for the recovery of gait after stroke. However, an impact study of the Predict REcovery Potential (PREP2) model for upper limb outcome post-stroke showed increased efficiency in terms of a reduced length of stay, a change in therapy content, as well as higher therapist confidence (30). Implementation of prediction models in clinical practice is challenging, as it requires a behavioral change of the treating therapists. Similarly, with outcome assessments (31), there are two stages that can be identified. First, the predictors have to be assessed and next, the information regarding the expected outcome has to be used in the rehabilitation of a patient (32). Studies on implementing prediction models in stroke are scarce. A study investigated the implementation of the PREP2 model and critical factors were the staff's level of knowledge regarding the model, their beliefs and self-efficacy, and the perceived benefits of applying the model (33). Predictors are most often assessed with standardized outcome measures, and from implementation research on stroke assessments (34), it is known that the willingness and ability to change are additionally hampered by the lack of familiarity with the assessments (31, 34), having difficulty with changing routines (34), time and equipment needed to perform assessments (31, 34), and the lack of support from the management staff or team members (31). Whether the assessment is performed is highly dependent on the inability of a patient to participate in the testing due to, for example, communication or cognitive deficits (31, 35). Based on the available implementation literature, an approach to implement the EPOS model for independent gait would be initiated by therapists themselves, having support from the management staff, having a close collaboration between researchers who developed the model and clinicians, providing ongoing education and ‘teaching on the job,' and having directions on important treatment foci (33).

Apart from continuing with the external validation of the EPOS model for independent gait in a more heterogeneous sample of patients and across different geographical regions and investigating the model's impact, an extension of the model applying a more fine-grained classification of FAC scores below 4 is needed. Even though a score below 4 means that the patient is not able to walk independently, the scores 0–3 cover a wide spectrum of walking abilities, ranging from 0 (i.e., not able to walk) to 3 (i.e., walking under supervision). Reaching a FAC of 2 (i.e., walking with intermittent or continuous light physical support for balance and/or coordination) or 3 could be highly valuable for patients and their caregivers and being able to make a more fine-grained prognosis with the following levels, (a) not regaining gait, (b) regaining gait with light physical support or supervision, and (c) regaining independent gait, would support clinicians in evidence-based goal setting and rehabilitation design.

Limitations of the current study includes a relatively low number of patients in the validation cohorts who were recruited in one center. Furthermore, there was a difference in the measurement time points of the predictors of the two validation cohorts that did not allow us to investigate the performance of a model on day 5. The inclusion of patients with first-ever stroke without large preexisting disability (mRS ≤2) hampers the generalizability of patients with recurrent strokes and those who were more disabled prior to their stroke. However, the only prestroke disability category missing is the mRS score of 3, as patients with a score of 4 or 5 cannot walk independently, and it is unlikely that they will regain this independence after stroke. Furthermore, patients in Cohort I were included earlier post-stroke than in the development cohort and one would assume that a large number of patients are unable to walk independently at this early time point after stroke. However, the inspection of the FAC scores at visit 2 showed that none of the patients had a score of 4 or 5. Last, we did not perform an update of the model, but this can be justified by the acceptable performance in the independent cohorts as well as the fact that the size of our cohorts would have resulted in an overfitted model (36).

## Conclusion

The external validation of the EPOS model for the outcome of independent gait 3 months after stroke was successful when sitting balance and strength of the paretic leg were assessed from day 3 onwards. With that, the model is generalizable to patients who had a first-ever ischemic or hemorrhagic stroke and were independent prior to their stroke. To increase the applicability of the EPOS model to a wider patient population, future multicenter validation studies should recruit a larger and more heterogeneous sample of patients, including those with recurrent strokes, neurological diseases, and higher levels of comorbidity. In addition, these validation studies should be performed in different geographical areas to extend the geographical validity of the EPOS model for independent gait.

## Data Availability Statement

The raw data supporting the conclusions of this article will be made available by the authors to qualified researchers, without undue reservation.

## Ethics Statement

The studies involving human participants were reviewed and approved by Cantonal Ethics Committee Zurich. The patients/participants provided their written informed consent to participate in this study.

## Author Contributions

JV and JH conceptualized and designed the study. JV, JP, and JH acquired data and interpreted the data. JV performed the analysis and drafted the manuscript. JP, JH, and AL revised the manuscript. All authors gave final approval of the version to be published and agreed to be accountable for all aspects of the work.

## Funding

The data collection for Cohort I was partly funded by Boehringer Ingelheim (contract number 43084008); the data collection for Cohort II was funded by the P&K Pühringer Foundation. The funders played no role in the design, conduct, analysis, and reporting of the work. For the rest of the work, no external funding was obtained.

## Conflict of Interest

The authors declare that the research was conducted in the absence of any commercial or financial relationships that could be construed as a potential conflict of interest.

## Publisher's Note

All claims expressed in this article are solely those of the authors and do not necessarily represent those of their affiliated organizations, or those of the publisher, the editors and the reviewers. Any product that may be evaluated in this article, or claim that may be made by its manufacturer, is not guaranteed or endorsed by the publisher.
