# Supplementary material for: External Validation of the Early Prediction of Functional Outcome After Stroke Prediction Model for Independent Gait at 3 Months After Stroke
Source: Front Neurol. 2022 May 2;13:797791. doi: 10.3389/fneur.2022.797791 (PMC9108182; doi:10.3389/fneur.2022.797791)
Supplement: Supplementary file 3 [file Data_Sheet_3.PDF]

# External validation of the EPOS prediction model for independent gait after stroke and extension to a 3-month endpoint

**Janne M. Veerbeek[1,2], Johannes Pohl[1,3], Jeremia P.O. Held[1,4], Andreas R. Luft[1,5]**

[1]Department of Neurology, University of Zurich and University Hospital Zurich, Zurich, Switzerland

[2]Neurocenter, Luzerner Kantonsspital, Lucerne, Switzerland

[3]Department of Rehabilitation Sciences, KU Leuven – University of Leuven, Leuven, Belgium

[4]Rehabilitation Center Triemli Zurich, Valens Clinics, Zurich, Switzerland

[5]cereneo, Center for Neurology and Rehabilitation, Vitznau, Switzerland

## *Supplementary Material*

1 Supplementary Tables and Figures ........ 2

1.1 Supplementary Table 1. Baseline Characteristics of Included Patients of the Development and Validation Cohorts ........ 2

1.2 Supplementary Table 2. Early Changes in Walking Ability in Cohort I ........ 4

1.3 Supplementary Table 3. Early Changes in Walking Ability in Cohort II ........ 5

1.4 Supplementary Table 4. Comparison of Patients With and Without Missing Data on Predictors and/ or Outcome ........ 6

1.5 Supplementary Table 3. Discrimination Analysis Based on Imputed and Raw Datasets ........ 7

1.6 Supplementary Figure 1. Calibration Plots based on the Raw Datasets ........ 8

2 Supplementary References ........ 9

# 1 Supplementary Tables and Figures

## 1.1 Supplementary Table 1. Baseline Characteristics of Included Patients of the Development and Validation Cohorts

| **Characteristic** | **Development cohort** (1) | **Validation cohort I** | | **Validation cohort II** | |
|---|---|---|---|---|---|
| | (N=154) | (N=39) | Missing data, N (%) | (N=78) | Missing data, N (%) |
| Age, years | 67.54 (14.15)* | 74 (69–77)† | 0 (0) | 69 (60–77)† | 0 (0) |
| Female, N (%) | 93 (60.4) | 13 (33.3) | 0 (0) | 29 (37.2) | 0 (0) |
| Affected hemisphere, left/ right, N (%) | 67 (43.5)/ 87 (56.5) | 13 (33.3)/ 26 (66.7) | 0 (0) | 37 (47.4)/ 41 (52.6) | 0 (0) |
| Type of stroke, N (%) | | | 0 (0) | | 0 (0) |
| Ischemic | 154 (100) | 39 (100) | | 60 (76.9) | |
| Hemorrhagic | 0 (0) | 0 (0) | | 28 (35.9) | |
| Bamford classification, N (%) | | | 0 (0) | | 0 (0) |
| LACS | 64 (41.6) | 16 (41) | | 31 (39.7) | |
| PACS | 57 (37) | 12 (30.8) | | 28 (35.9) | |
| TACS | 33 (21.4) | 11 (28.2) | | 19 (24.4) | |
| Thrombolysis, yes, N (%) | 44 (28.6) | 15 (38.5) | 0 (0) | 14 (17.9) | 0 (0) |
| Thrombectomy, yes, N (%) | N/A | 16 (41) | 0 (0) | 25 (32.1) | 0 (0) |
| Prestroke mRS (0–5)† | N/R | 0 (0–0) | 0 (0) | 0 (0–0) | 0 (0) |
| Time poststroke (days) | | | | | |
| Model day 2 | 2.24 (1.32)* | 1 (1–1)† | 0 (0) | 3 (2–4)† | 0 (0) |
| Model day 9 | 9.00 (1.84)* | 8 (7–8)† | 0 (0) | 9 (8–10)† | 4 (5.1) |
| Clinical scales baseline | | | | | |
| NIHSS (0–42)† | N/R | 9 (5.5–13.5) | 0 (0) | 8 (5–12) | 0 (0) |
| Cognitive disturbance, yes, N (%) | | | | | |
| Inattention | 75 (48.7) | 18 (46.2) | 0 (0) | 24 (30.8) | 0 (0) |
| Disorientation | 37 (23.7) | 14 (35.9) | 0 (0) | 22 (28.2) | 0 (0) |
| Sensation deficits, yes, N (%) | 97 (63) | 21 (53.8) | 0 (0) | 38 (48.7) | 0 (0) |
| Visual impairment, yes, N (%) | | | | | |
| Hemianopia | 52 (33.8) | 6 (15.4) | 0 (0) | 25 (32.1) | 0 (0) |

| | | | | | |
|---|---|---|---|---|---|
| Deviation conjugee | 45 (29.2) | 13 (33.3) | 0 (0) | 17 (21.8) | 0 (0) |
| MI lower extremity (0–100)† | 44.50 (9–69.75) | 37 (20.25–60.5) | 1 (2.6) | 42 (28–64) | 0 (0) |
| MI upper extremity (0–100)† | 33 (33–65) | 39 (4.5–61) | 0 (0) | 47 (15–61) | 0 (0) |
| FMMA lower extremity (0–34) | 17 (6.75–25.25) | N/A | | N/A | |
| FMMA upper extremity (0–66)† | 12 (4–43.25) | 10.5 (4-23.5) | 1 (2.6) | 19.5 (6–31) | 0 (0) |
| FAC (0–5)† | 0 (0–2) | 0 (0–0) | 0 (0) | 0 (0–2) | 0 (0) |
| mRS (0–5)† | N/A | 5 (4–5) | 0 (0) | 5 (4–5) | 0 (0) |
| BI (0–20)† | 5 (1–23) | N/A | | N/A | |
| Predictors | | | | | |
| Model day 2 | | | | | |
| Sitting balance, yes, N (%) | 104 (67.5) | 17 (43.6) | 0 (0) | 47 (60.3) | 0 (0) |
| Strength leg, yes, N (%) | N/R | 28 (71.8) | 1 (2.6) | 60 (76.9) | 0 (0) |
| Model day 9 | | | | | |
| Sitting balance, yes, N (%) | N/R | 29 (78.4) | 2 (5.1) | 58 (78.4) | 4 (5.1) |
| Strength leg, yes, N (%) | N/R | 30 (83.3) | 3 (7.7) | 62 (83.8) | 4 (5.1) |
| Outcome | | | | | |
| FAC ≥4, N (%) | 122 (79) | 28 (71.8) | 0 (0) | 58 (74.4) | 0 (0) |
| FAC (0–5)† | | | | | |
| subgroup <4 | N/R | 1 (0.5–3) | | 1.5 (0–3) | |
| subgroup ≥4 | N/R | 5 (4–5) | | 5 (4–5) | |

Legend: *, mean (standard deviation); †, median (interquartile range); ARAT, Action Research Arm Test; BI, Barthel Index; FAC, Functional Ambulation Categories; FMMA, Fugl-Meyer Motor Assessment; LACS, Lacunar Stroke; MI, Motricity Index; mRS, modified Rankin Scale; N, Number; N/A, Not Applicable; N/R, Not Reported; NIHSS, National Institutes of Health Stroke Scale; PACS, Partial Anterior Circulation Stroke; TACS, Total Anterior Circulation Stroke.

## 1.2 Supplementary Table 2. Early Changes in Walking Ability in Cohort I

| | | FAC | | | | | | |
|---|---|---|---|---|---|---|---|---|
| | **Day 8** / **Day 1** | **0** | **1** | **2** | **3** | **4** | **5** | **Missing data** |
| **FAC** | **0** | 15 | 6 | 5 | 0 | 1 | 2 | 2 |
| | **1** | 0 | 0 | 0 | 1 | 2 | 0 | 0 |
| | **2** | 0 | 0 | 0 | 0 | 2 | 1 | 0 |
| | **3** | 0 | 0 | 0 | 0 | 1 | 1 | 0 |
| | **4** | 0 | 0 | 0 | 0 | 0 | 0 | 0 |
| | **5** | 0 | 0 | 0 | 0 | 0 | 0 | 0 |

Legend: FAC, Functional Ambulation Categories. Note that this information was not provided by the development study.

## 1.3 Supplementary Table 3. Early Changes in Walking Ability in Cohort II

| | | FAC | | | | | | |
|---|---|---|---|---|---|---|---|---|
| | Day 9<br>Day 3 | 0 | 1 | 2 | 3 | 4 | 5 | Missing data |
| FAC | 0 | 23 | 3 | 7 | 6 | 1 | 1 | 2 |
| | 1 | 0 | 2 | 1 | 2 | 2 | 0 | 0 |
| | 2 | 1 | 1 | 4 | 1 | 6 | 4 | 2 |
| | 3 | 0 | 0 | 0 | 1 | 3 | 5 | 0 |
| | 4 | 0 | 0 | 0 | 0 | 0 | 0 | 0 |
| | 5 | 0 | 0 | 0 | 0 | 0 | 0 | 0 |

Legend: FAC, Functional Ambulation Categories. Note that this information was not provided by the development study.

## 1.4 Supplementary Table 4. Comparison of Patients With and Without Missing Data on Predictors and/ or Outcome

| Characteristic | Validation cohort I | Validation cohort II |
|---|---|---|
| | **P-value** | **P-value** |
| Age, years | 0.938 | 1.000 |
| Gender, female/ male | 1.000 | 1.000 |
| Affected hemisphere, left/ right | 0.571 | 0.661 |
| Bamford classification, LACS/ PACS/ TACS | 0.509 | 0.602 |
| Thrombolysis, yes/ no | 0.713 | 0.745 |
| Thrombectomy, yes/ no | 1.000 | 1.000 |
| Time poststroke model day 2 | 0.837 | 0.856 |
| Clinical scales baseline | | |
| NIHSS (0–42) | 0.918 | 0.604 |
| FAC (0–5) | 0.398 | 0.347 |
| mRS (0–5) | 0.203 | 0.144 |
| Predictors model day 2 | | |
| Sitting balance, yes/ no | 1.000 | 1.000 |
| Strength paretic leg, yes/ no | 1.000 | 1.000 |

Legend: Mann-Whitney U for ordinal data and Chi-square test for binary data; FAC, Functional Ambulation Categories; LACS, Lacunar Stroke; MI, Motricity Index; mRS, modified Rankin Scale; NIHSS, National Institutes of Health Stroke Scale; PACS, Partial Anterior Circulation Stroke; TACS, Total Anterior Circulation Stroke.

## 1.5 Supplementary Table 3. Discrimination Analysis Based on Imputed and Raw Datasets

| | **Validation cohort I** | | **Validation cohort II** | |
|---|---|---|---|---|
| | Imputed data | Raw data | Imputed data | Raw data |
| Model day 2 | N=39 | N=38 | N=78 | N=78 |
| Accuracy (95% CI) | 0.641 | 0.632 | 0.833 | 0.833 |
| Sensitivity | 0.786 | 0.778 | 0.931 | 0.931 |
| Specificity | 0.273 | 0.273 | 0.550 | 0.550 |
| Positive predictive value | 0.733 | 0.724 | 0.857 | 0.857 |
| Negative predictive value | 0.333 | 0.333 | 0.733 | 0.733 |
| No information rate | 0.718 | 0.711 | 0.744 | 0.744 |
| P-Value [Acc > NIR] | 0.892 | 0.893 | 0.041 | 0.041 |
| AUC (95% CI) | 0.675 (0.510, 0.841) | 0.667 (0.500, 0.834) | 0.801 (0.684, 0.918) | 0.801 (0.684, 0.918) |
| Model day 9 | N=39 | N=36 | N=78 | N=76 |
| Accuracy (95% CI) | 0.897 | 0.917 | 0.859 | 0.865 |
| Sensitivity | 0.964 | 0.963 | 0.931 | 0.929 |
| Specificity | 0.727 | 0.778 | 0.650 | 0.667 |
| Positive predictive value | 0.900 | 0.929 | 0.885 | 0.897 |
| Negative predictive value | 0.889 | 0.875 | 0.765 | 0.750 |
| No information rate | 0.718 | 0.750 | 0.744 | 0.757 |
| P-Value [Acc > NIR] | 0.007 | 0.011 | 0.010 | 0.017 |
| AUC (95% CI) | 0.921 (0.811, 1.000) | 0.912 (0.788, 1.000) | 0.846 (0.741, 0.951) | 0.832 (0.718, 0.947) |

Legend: Legend: Acc, Accuracy; AUC, Area Under the Curve; CI, Confidence Interval; N/R, Not Reported; NIR, No Information Rate.

## 1.6 Supplementary Figure 1. Calibration Plots based on the Raw Datasets

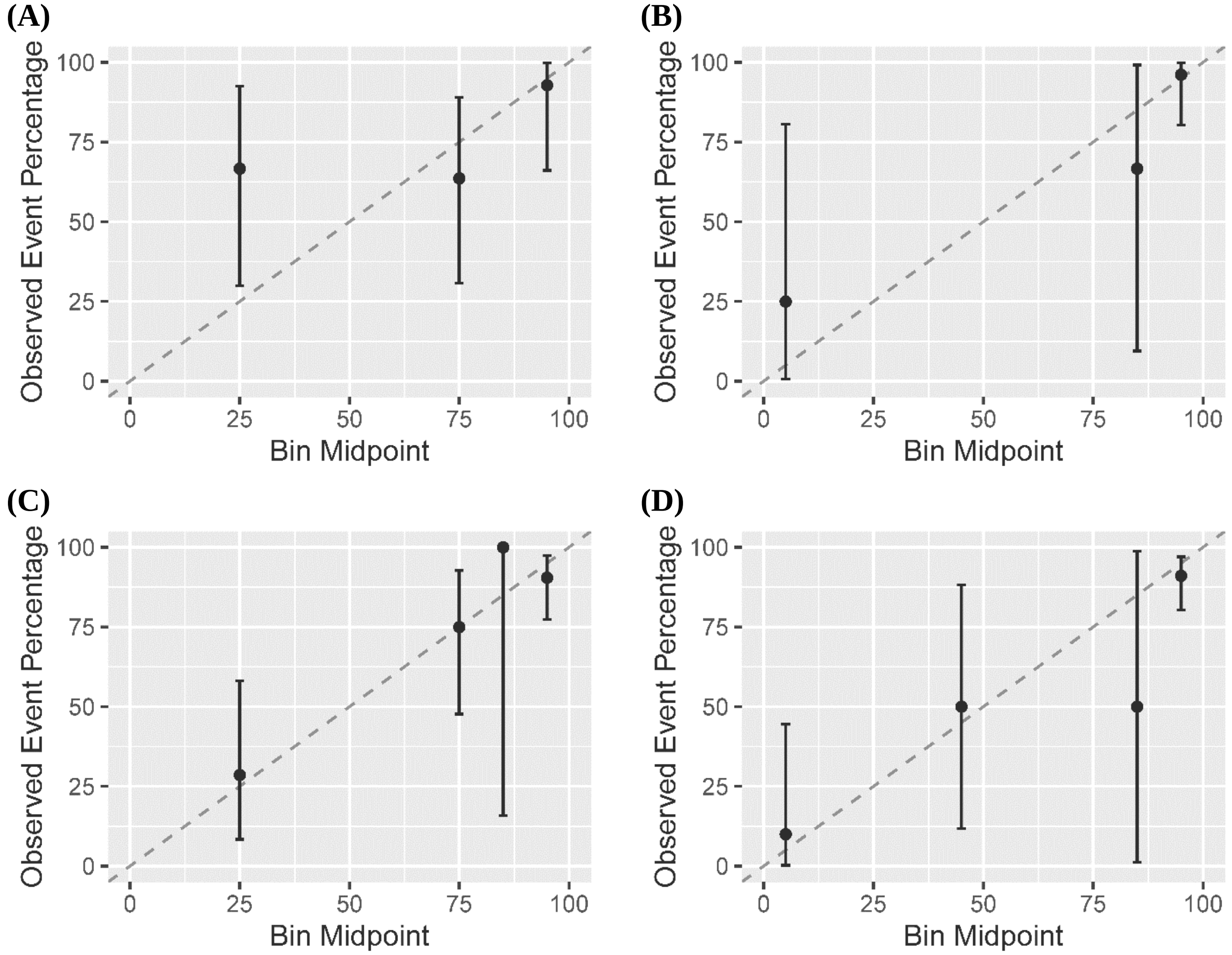


Legend: A calibration plots show the agreement between the predicted probabilities based on the EPOS model for independent gait on the x-axis and the in the validation cohorts observed probabilities on the y-axis. The closer the points are to the plotted diagonal line, the better the calibration. Points above the diagonal line indicate the model is rather pessimistic, points below the line indicate the model is rather optimistic. Confidence intervals overlapping with the diagonal $45^{o}$ line indicate no significant difference between predicted and observed probabilities. Cohort 1: **(A)** EPOS model day 2, measured at day 1 poststroke; and **(B)** EPOS model day 9, measured at day 8 poststroke. Cohort 2: **(C)** EPOS model day 2, measured at day 3 poststroke; and **(D)** EPOS model day 9, measured at day 9 poststroke.

## 2 Supplementary References

1. Veerbeek JM, van Wegen EE, Harmeling-van der Wel BC, Kwakkel G. Is accurate prediction of gait in nonambulatory stroke patients possible within 72 hours poststroke? The EPOS study. Neurorehabil Neural Repair. (2011) 25:268-74. doi: 10.1177/1545968310384271
